# Inducement factor of talent agglomeration in the manufacturing industrial sector: A survey on the readiness of Industry 4.0 adoption

**DOI:** 10.1371/journal.pone.0263783

**Published:** 2023-10-05

**Authors:** Yanchao Feng

**Affiliations:** College of Management and Economics, Tianjin University, Tianjin, China; Sri Eshwar College of Engineering, INDIA

## Abstract

China’s economy has progressed from a rapid growth phase to one of high-quality development and innovation. Industry 4.0 manufacturing technology and processes include cyber-physical systems (CPS), Industrial Internet of Things (IIOT), Cognitive Computing and Artificial Intelligence (CCAI) as advancements in computerization and information exchange the relevant variables data, and a survey questionnaire are used to accumulate three-year data from 2017 to 2019. The Structured Equation Modeling (SEM), analytic hierarchy process (AHP), and mediating variable in a SOBEL test are applied. The results show that Industry 4.0 is the primary practical corridor to official and familiar in sequence substitute policy and collaboration for talent agglomeration on research projects. It lowers the fixed price of human capital and significant factors active long-term innovation and profit at the end of the inferential test results. Hypotheses findings show that the associations between dependent and independent variables are essential, and latent variables GFI, CFI, TLI, and IFI have acceptable values. CMINDF and RMR fulfill the fit criteria and results will assist managers and policymakers in spotting talent agglomeration activities implemented to increase manufacturing businesses’ readiness to reap the most benefits from Industry 4.0 adoption.

## 1. Introduction

Large economies, such as the United States, Europe, China, and Japan, rely heavily on manufacturing. Changing demographics, globalization, resource shortages, climate change problems, and mass customization are all megatrends that threaten manufacturing’s future [1]. It is critical to obtain innovations in manufacturing techniques and systems daily, and worker talent agglomeration is a key component of the research and development sector for advancing manufacturing processes. In recent years, talent agglomeration has emerged as a significant source and endogenous element of social-economic growth in China, with a scarcity of talent. The objective of the study is to determine the talent agglomeration factors in the manufacturing industrial sectors of China especially to determine the benefits of adoption of Industry 4.0 in these manufacturing industries. For China to accomplish talent agglomeration, industrial upgrading, and quality manufacturing development, Industry 4.0 is critical. The problem statement of this study is the driving force of Industry 4.0 as a talent agglomeration in China’s manufacturing industry is investigated in the influence of Industry 4.0 on firm performance. Industry 4.0 is an acronym for the fourth industrial revolution, which continues the third and aims to achieve a high level of automation in manufacturing industries [2]. Small and medium-sized companies (SMEs) constitute the cornerstone for manufacturing sectors, the backbone of big economies like the United States, Europe, China, and Japan. Small and medium-sized businesses are more adaptable to new technologies and specialized markets because of talent agglomeration [3]. The advantages of supporting Industry 4.0 include that it encourages smart production from various perspectives, allowing for rapid talent agglomeration and improved supply chain performance. A new kind of industrial organization tightly connected online and offline is the talent agglomeration of creative industries based on the Internet and digital technologies [4]. On through crowd intelligence, crowd fundraising, and crowd sourcing on the Internet, creative organizations can get creative inspiration, financial support, and talent agglomeration assistance [5]. The Nobel Prizes in Chemistry, Medicine, Physics, and Economics, as well as the Nobel Memorial Prize in Economics, are all excellent examples such as (i) the exponential rate of technical progress; (ii) the breadth and depth of technological innovation, and (iii) the magnitude of the effect throughout the whole system, which affects firms, industries, and entire countries indicate that Industry 4.0’s disruptive impacts are seen at all levels [6]. Bai and Tong [7] use industry-level data to show that the degree of industrial agglomeration in China decreased, and then increased, from 1985 to 1997 [8]. China’s industrial production exhibited considerable agglomeration toward the coastal area. However, the as of immobility of natural resources underpins the drive of industrial agglomeration [9]. Talent agglomeration is critical for Chinese manufacturing industries’ capacity. The advanced talent agglomeration will affect the Chinese adoption of Industry 4.0 manufacturing capabilities, human capital, and R&D commitment. The major contribution of this study is to determine the rule of Industry 4.0 to promote talent agglomeration in Chinese manufacturing industries by applying the structured equation modeling (SEM), analytic hierarchy process (AHP), convergent validity test, corporate finance institute (CFI) model and questionnaire data is analyzed for skewness and kurtosis. This study aims to look at Industry 4.0’s strategic response to talent agglomeration in Chinese manufacturing industries and the essential variables that will help it succeed. Human capital in Chinese cities significantly influences the site selection of utility-maximizing high-skill employees, suggesting a trend of human capital agglomeration in China’s cities [10]. In the realm of Industry 4.0, China is currently the world’s top producer in a variety of industries [11]. China’s industrial sector was experiencing problems such as growing labour costs, environmental and resource constraints, and a deteriorating trading climate [12]. China has actively promoted Industry 4.0 to move manufacturing from "Big" to "Strong." China and Germany announced a collaborative action plan titled "Shaping innovation together" in October 2014 to strengthen bilateral collaboration in the promotion of Industry 4.0 [13]. In the age of globalization, the adoption of industry 4.0 is one of the drivers that allow industries to reach the necessary output level while lowering production costs. The agglomeration of innovative talent and human capital has a major influence on manufacturing industrial innovation capacity at the knowledge innovation stage. Several studies relating to the industrial sector have been conducted in China. However, there was no study on industry 4.0, one of the most pressing concerns in the worldwide market. We recognize the need for a global and local innovation sector that promotes industry 4.0 principles and talent agglomeration in the Chinese manufacturing Industrial sector.

The following is a remaining parts of the paper’s structure. Section 2 describes the major theoretical part known as literature review. Section 3 demonstrates the innovation talent agglomeration methodology and research design; Section 4 results analyses and discusses the empirical results; conducts the robustness test; and Section 5 conclusion with future suggestions and recommendations.

## 2. Literature review

This investigation focuses on the aggregation of cutting-edge inventive abilities in China’s innovative industry 4.0. Industry 4.0, which focuses on data innovation sectors like data transmission, electronic administrations, and programming [14], is one of the world’s most dynamic businesses worldwide. Industry 4.0 is focused on combining innovation and cycles, such as existing digital frameworks, Web of things, and current Web of things, as well as personal computers and information sharing [15]. Some ideas that describe how people react to new technology include the Doorman’s worth chain and the innovation system climate [16]. Individuals with cutting-edge development gifts have an undeniable personal quality, advanced science and innovation knowledge, proficient abilities, and a strong imaginative capacity [17]. Existing research [18] has tremendously endorsed the significant advantages of capability agglomeration on financial growth and current events, but a continual increase in scale isn’t always beneficial to monetary outcomes. It also has negative consequences, such as increased traffic, pollution, and a mismatch between the size of development clusters and territorial advancement capability [19]. [20] depicts total capability through Industry 4.0 as a way to investigate cultural acceptability of innovation by defeating some of augmented reality innovation requirements and utilizing it as anything other than a distant upkeep administration, once again introducing A.R. as a reasonable industry apparatus. [21] presented that Industry 4.0 is critical in the manufacturing industry for talent agglomeration. Cyber-physical systems and digital integration of production and business processes are two aspects of advanced manufacturing strategies from a technological standpoint. In the industrial environment, Industry 4.0 is a fundamental and novel concept [22]. Between 2010 and 2020, China’s creative crowd sourcing service platform Yipin Witkey registered over 22 million users, opened over 300 types of creative services, and successfully solved the creative needs of 9.8 million businesses. A new sort of creative sector growth model is evolving [23]. Using the network culture industry [24] discovered that the effect of citizens, technology, government, and other variables on the network culture industry ecosystem has altered compared to conventional geographic agglomeration [25]. The Chinese government plays a minor role in encouraging businesses to implement Industry 4.0, and accusations that China has an unfair industrial policy are unfounded. Industry 4.0 gives China more opportunities to improve its talent agglomeration capability in the manufacturing industries [26]. China’s government established the "Made-in-China 2025" plan based on Industry 4.0, which focuses on integrating existing sectors and strengthening China’s manufacturing position in the global value chain [27]. The terms Industry 4.0, smart manufacturing, and Made in China 2025 are frequently used interchangeably in China. Manufacturing businesses in China have also been vocal proponents of Industry 4.0. [28] looked at how Apple’s iPad and iPhone capture value in global networks and discovered that sold in the United States adds $229 to $275 to the U.S.–China trade deficit, it only adds $10 or less in direct labour wages to China’s workers. Furthermore, manufacturing progress frequently results in issues like pollution and carbon emissions [29]. High-skill individuals attract a larger labour pool with more skill-demanding employment [30], and the motives of this human capital agglomeration have been thoroughly discussed. As an alternative source of agglomeration economics, knowledge spillover contributes to the geographical concentration of human capital [31]. The third main view is based on spatial sorting by personal capacity [32]. According to [33], China’s state-owned companies have been utilized to preserve social stability and serve other social, philanthropic goals. It creates a variable share of state-owned production for each three-digit industry, and outsiders have been regarded as a significant factor for industrial agglomeration [34]. Local protectionism is not directly measured except in a few rare situations must deduce their existence through indirect ways that capture these externalities. To describe one of the ten future initiatives intended to underlie the German government’s approach to industrial modernization, the High Tech 2020 Strategy [35], Industry 4.0 developed as a policy idea. [36] stated various inconsistence to refer to technologies and processes generally classified under the label ’Industry 4.0. In reality, the size of the development is a measurement of the distance from the technological frontier, which explains the productivity gap and lack of convergence and provides a case for industrial policies [37]. Industries 4.0 have enormous potential, and fundamental changes in organization, market modalities, and production technologies will result in many economic and social prospects [38]. [39] created a maturity model framework that is split into nine dimensions and 62 maturity items in research. The German government recommends implementing 4.0 and use scientific studies, reports, and works for this middle age item and various dimension formulation [40].

## 3. Research methodology

### 3.1 Data collection

Senior managers and technical executives in automotive firms in charge of production, information management, and maintenance are the study’s target Agglomeration population—the information related to these firms collected from the website Chinese Ministry of Commerce, http://english.mofcom.gov.cn. We developed the survey instrument questionnaire The need weight of each sub-pointer in the connected marker framework is controlled by duplicating the neighborhood weight by comparing classification weight, and the worldwide weight is determined by increasing the nearby weight by the relating classification weight. Questionnaire consists of 34 questions, 4 categories, and 15 sub-indicators of 1–5 ranked were mentioned in Table 1, and in Table 14 which is shown in S1 Appendix together the required information from the professionals and managers of manufacturing businesses. Most employees at the firm are aware of talent agglomeration and Industry 4.0, according to data collected between 2017 and 2019. Out of 750 distributed questionnaires 525 respondents filled out the questionnaires consist of from the manufacturing industries, resulting in the following data: 255 manufacturing industries.

**Table 1:** Factors that influence talent agglomeration indicators.

Indicators	Sources
Ecological locality	Mozzato et al. (2018) [48]
Livelihood situations	Ajmal et al. (2010) [49]
Standing	Oketch, 2006 [50]; Florida, (2002) [51]
Manager Concentration	Barrow and Mosley (2011) [52]
Promotion	Combes and Gobillon (2015) [53]
Employment Contentment	Carlino and Kerr (2015) [54]
Talent Estimation classification	Schein, 1996; [55]
Work Realization	Faggio et al. (2017) [56]
Elasticity	Wang and Wang, 2014) [57]
Livelihood civilization	Docquier and Rapoport, 2012) [58]
commercial operation	Chaubey and Sahoo (2019) [59]
Principles of utilization	Docquier and Rapoport, (2012) [58]
Managerial Attitude	Fang et al. (2014) [60]
GDP growth	Meyers et al. (2019) [61]
Team teamwork	Hogg and Turner (1985) [62]
monetary growth intensity	Cronbach, (1975) [63]
Income rank	Gibson et al. (2011) [64]
company target	Collings and Mellahi (2009) [65]
Flaxen Routine Appraisal	Steinbaum et al. (2017) [66]
corporation visualization	Errata et al. (2018) [67]
Research & development expenditures	Kerr et al. (2017) [68]

**Fig 1:**
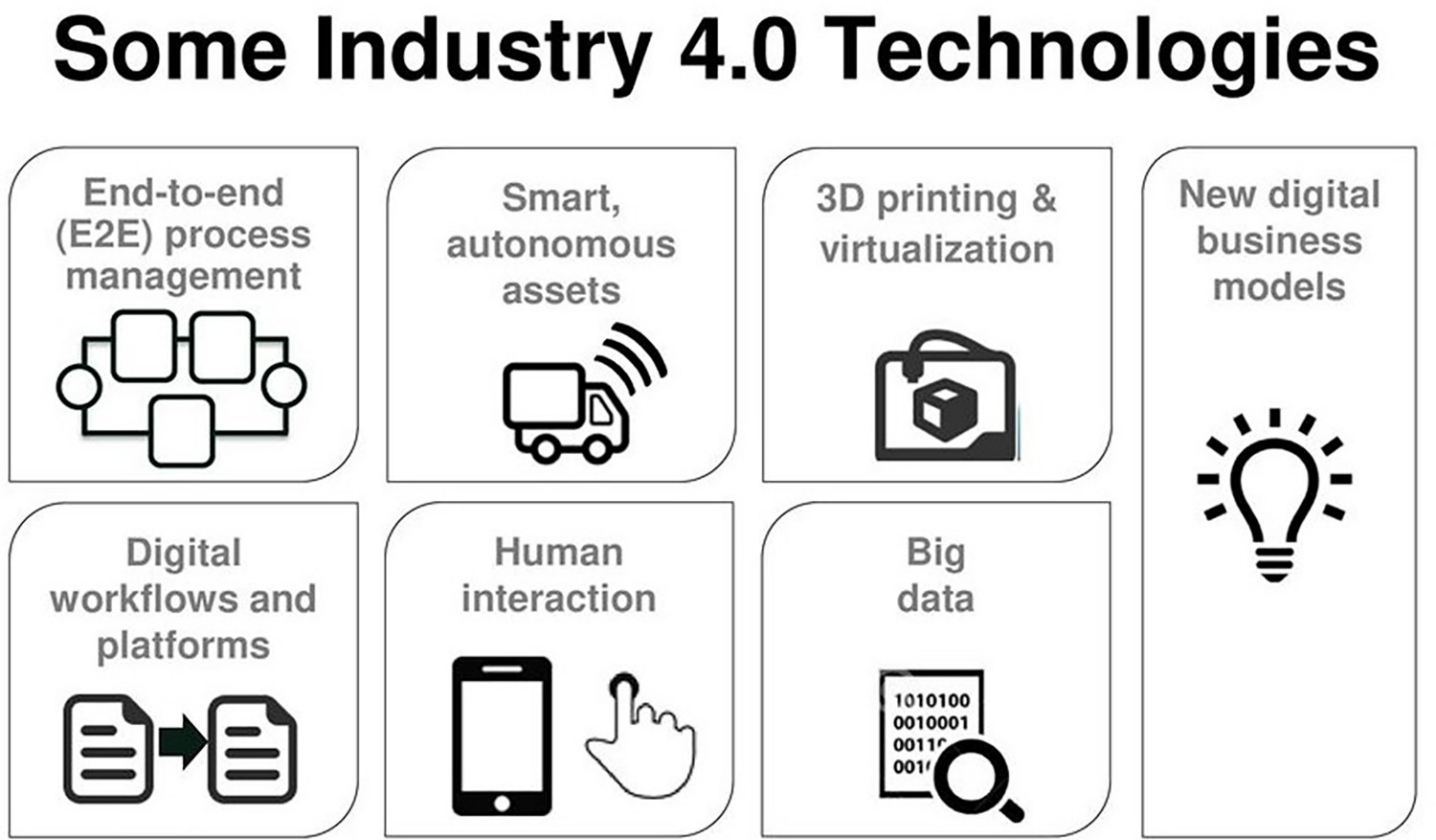
Shows the some Industry 4.0 technologies complete process. Source: DOI: Prof Dr. ANDRE LUDWIG https://slideplayer.com.

We divide the factors into three key areas to make a nation excellent in manufacturing in a digital environment, the twenty-first century, with the availability of data: Manufacturing industries as a proportion of GDP comprise (i) manufacturing capabilities, (ii) research and development, (iii) human capital, (iv) products are included as explained in flowchart in Fig 1.

### 3.2 Hypothesis development

We construct five hypotheses based on five dimensions, their related literature, and the conceptual framework described above:

**H**_**01**_: People, consumers, and culture have little bearing on talent agglomeration innovation decision-making.

**H**_**02**_: Strategy and leadership have little impact on talent agglomeration innovation decision-making.

**H**_**03**_: Governance and operations have little impact on talent agglomeration innovative decision-making.

**H**_**04**_: Government initiatives help to balance the importance of people, consumers, and culture in talent agglomeration innovation decision-making.

**H**_**05**_: Interventions by the government modify the relationship between talent agglomeration innovation decision-making and strategy and leadership.

**H**_**06**_: Government actions influence the relationship between talent agglomeration innovation governance and operations.

### 3.3 Data analysis techniques

Structured Equation Modeling (SEM) was employed to test the hypothesis since it allows the researcher to simulate connections between numerous predictors and determinant factors. Other widely acknowledged assets are accessible global fit tests, and the SEM typically comprises of measurement theory and equation model [41], and equations are as follows:

Y=Δyρ+ε (i)

X=Δxφ+∈ (ii)

And, the structural equation model is specified as:

ρ=α+βρ+λφ+φ (iii)

Where, Y = Resultant Variables, X = Participation Variables, Δy = latent variables, Δx = latent variables, ε = error, ϵ = error term, ρ = latent variables, φ = latent variables, α = vector of intercepts, and β = matrix of coefficient.

### 3.4 Analytic hierarchy process

The analytic hierarchy process (AHP) is a systematic approach based on mathematics and psychology for organizing and evaluating complicated choices. It was created in the 1970s by Thomas L. Saaty, (1970) collaborated with Earnest Forman to create Expert Choice in 1983, and has since been extensively explored and modified. It is a precise method for calculating the weights of choice criteria. Pair-wise comparisons are used to assess the relative magnitudes of variables based on individual experts’ experiences. Each responder must rate the relative significance of the two topics using a specially constructed questionnaire (note that whereas most polls use a five-point Likert scale, AHP’s questionnaire uses a 9-to-1-to-9 scale [42]. The method is repeated for all factors, starting at the top of the hierarchy and moving down. Then several preference squares with their associated eigenvectors are created as shown in Eq (iv) is a generic matrix:

$$S≅\Delta \beta_{ij}$$ (iv)

A one-to-nine scale is used influencing factors of HTIT agglomeration indicators to compare alternatives as indicated in Table 2. When the hierarchical method could be followed and proven fairly dependable, this scale was frequently employed. The five categories on the scale are making things even easier, equally significant, and weakly significant, enormously significant, very strongly significant, and more significant.

**Table 2:** Influencing factors of HTIT agglomeration indicators (after expert interviews).

Indicators	Sub-indicators	The operational definition of sub-indicators
Individual Incentive	Continuing Growth	Involvement and activities are geared toward improving professional development, including promotion, reputation, participation in professional organizations and skill-based training
	Sense of working accomplishment	Fulfillment of the possibilities of one’s character or personality, including self-fulfillment, self-worth and self-actualization
	Self-realization pursuit	
Organizational	Flexible Specialization	Corporate governance mechanism view as a response to a change that improves the project schedule and arises with sustainable performance in the technologies of communication, including foreseeable growth of the company, foreseeable increase of market share, flexible management and patents
Incentive	R&D investment	Spending on research and development including R&D expenditure, R&D proportion and technological innovation support
Social Incentive	Talent Evaluation System	Measurable and qualitative evaluation of key performance indicators (KPIs) including the propensity of R&D and innovative achievement and fair performance assessment
	Cultural Background	Living customs, values, human environment and innovative and entrepreneurial atmosphere
	Gender and Ethnic Equality	
	Economic Development level	The standard of a nation or a region improves the economic, political and social well-being of its people, including GDP, standards of consumption and average salary levels
	Industrial Cluster	The proportion and numbers of similar enterprises in the same industry in a relatively concentrated place

## 4. Results and discussions

The last score was determined utilizing the average significance score of 63 specialists, just as specific decisions’ and the calculation of nearby loads and factor requesting levels, as shown in Table 3. The average load of each sub-pointer in every classification is included to as neighborhood weight for the subsequent norm the average arbitrary consistency record.

**Table 3:** Local weights and the ranking levels of factors.

Category	Weight	Sub-indicator	Local Weight	Rank
Entity Inducement	0.76	Employment Contentment	0.43	1
		Intellect of working accomplishment	0.53	2
		long-term development	0.45	3
		Self-realization follow	0.34	4
		wellbeing Successions	0.39	5
Managerial Inducement	0.48	Interpersonal affiliation	0.62	1
		secretarial civilization	0.34	2
		elastic specialty	0.67	3
		R&D venture	0.31	4
		Talent assessment classification	0.17	5
Societal Inducement	0.29	Gender and racial parity	0.64	1
		Political classification	0.69	2
		educational environment	0.56	3
		manufacturing gather	0.26	4
		financial growth intensity	0.12	5

Consequent average is the regularity directory of the identical organizes arbitrary decisions matrix, disadvantage, the consistency index grows significantly to an optimal level as N enhances. In conclusion, C.S regularity proportion is calculated the significance of C.R. is less than 0.1, the reliability test is approved. Table 4 shows that on an international level, the consistency ratios of feature weights and rankings are all less than 0.1, suggesting that the judging matrix satisfies the standards.

**Table 4:** Global weights and the ranking levels of factors.

Rank	Global ranking	Sub-indicator	Global weight
1	Job satisfaction	0.17	Individual incentive
2	Sense of working accomplishment	0.15	Individual incentive
3	Interpersonal relationship	0.12	Organizational incentive
4	Continuing growth	0.08	Individual incentive
5	Self-realization pursue	0.08	Individual incentive
6	Organizational culture	0.08	Organizational incentive
7	Welfare benefit	0.07	Individual incentive
8	Gender and ethnic equality	0.07	Social incentive
9	Flexible specialization	0.05	Organizational incentive
10	Political system	0.05	Social incentive
11	R&D investment	0.03	Organizational incentive
12	Talent evaluation system	0.03	Organizational incentive
13	Industrial cluster	0.02	Social incentive
14	Cultural background	0.02	Social incentive
15	Economic development level	0.01	Social incentive

This study adds to the current body of information on Industry 4.0 by looking at the factors influencing the adoption of challenging technologies in automotive firms. Several intriguing claims can be deduced from the results supported by the framework and technical measurement; the results demonstrated the importance of method growth.

### 4.1 Convergent validity test

The findings of the validation and measurement and the structured SEM process are all given. First, we examined the factor loading to determine reliability; factor loads > 0.83 applied, and those greater than 0.64 were benchmarked [43]. For the Convergent Validity test, the average variance extracted (AVE) was employed (CV). The degree to which two conceptually comparable tests of constructs are now connected is referred to as CV, and Composite Reliability (C.R) must be more than 0.5 and more significant than AVE [44]. The entire structure satisfies the CV requirements. A concept test’s discriminates validity (D.R) indicates that it is not as closely related to other measures measuring potentially different ideas. They must ensure specific conditions for data validation, which are C.R > AVE, AVE > MSV, AVE > ASV, and AVE > r. (correlation), all results of correlation analysis after running are shown in the Table 5.

**Table 5:** Represents the C.R, AVE and correlations between all variables results.

Construct	CR	AVE	MOI	NCC	MAG	TOP	MDM
MOI	0.959	0.839	0.921				
NCC	0.903	0.794	0.598	0.876			
MAG	0.7985	0.657	0.783	0.795	0.825		
AOP	0.891	0.765	0.768	0.778	0.629	0.827	
MDM	0.782	0.932	0.623	0.821	0.565	0.625	0.894

They must ensure specific requirements for data validation, such as AVE> 0.5, C.R.>0.83, C.R.>AVE, AVE>MSV, AVE>ASV, and AVE> r. (correlation). When these requirements are compared to the outcome mentioned above, the data meets all validity criteria. As a result, we may conclude that our model’s validity remains unaffected.

### 4.2 Descriptive statistics analysis

The inferential analysis uses a random sample of data from a population to describe and settle on the people. Furthermore, inferential analysis aids in the interpretation of sample data so that potential fairness may be anticipated and quantified. Essentially, our survey data is used to determine approximate population numbers. The statistics used to assess population figures, on the other hand, are primarily based on our survey data [45]. Descriptive statistics, Explanatory Factor Analysis (EFA), Confirmatory Factor Analysis (CFA), Path Analysis, and Mediation Analysis are all included in this section. The preliminary results of arithmetic mean, median, standard deviation, skewness, kurtosis, and other tests that explain the data’s initial results and suitability for critical analysis are shown in Table 6 as summary statistics.

**Table 6:** Summary statistics.

Construct	Variables	N	Min.	Max.	Mean	Std. deviation	Skewness	Kurtosis
Nation, consumers, and civilization	NC1	525	1	5	1.96	2.345	1.578	3.435
	NC2	525	1	5	3.35	2.455	0.789	-0.765
	NC9	525	1	5	4.56	3.561	0.578	-0.897
	NC9	525	1	5	2.02	1.126	1.096	0. 129
Method and Guidance	MG1	525	1	5	1.92	. 606	2.699	9.006
	MG2	525	1	5	1.94	. 699	1.999	9.699
	MG9	525	1	5	1.57	. 669	2.909	6.199
Authority and Procedure	AP1	525	1	5	1.87	. 905	2.566	8.345
	AP2	525	1	5	1.96	0.7328	2.457	9.342
	AP9	525	1	5	1.56	0.577	3.278	9.234
	AP9	525	1	9	2.12	0.698	2.871	2.121
Supervision Involvement	SI1	525	1	5	2.32	0.923	1.255	1.897
	SI2	525	1	5	2.56	0.654	0.754	2.167
	SI9	525	1	5	2.92	0.789	. 566	0.943
	SI9	525	1	5	1.99	. 929	1.199	2.199
Modernization and Decision Making	MD1	525	1	5	2.61	1.076	. 296	-1.876
	MD2	525	1	5	2.59	0.789	. 516	-0.7328
	MD9	525	1	5	2.29	1.589	0.789	-0.734
	MD9	525	1	5	2.22	0.934	0.876	-0.0634
Valid N (series)		525						

Source: field survey

The descriptive statistics for the variables utilized in the regression estimation as causes and indicators show Mean, standard deviations, skewness, and kurtosis. The mean of the replies in the 525 samples of 750 questionnaires ranged from 1.32 to 2.58, indicating that the industries are unfamiliar with various actions to promote industry I4.0.

### 4.3 Corporate financing intuitional test analysis results

The sample size, normalcy, missing values, and multi-co-linearity assumptions are evaluated using a structural equation model (SEM). Questionnaire data is analyzed for skewness and kurtosis to assess normality between the (-1) and (+1) range. In Table 7, the data set contained no outliers, according to the frequency count, and correlation analysis was employed to check for multi-co-linearity.

**Table 7:** The results of CFI model values.

Measurement Items	Constructs	CFI	R2	Factor Loading	Cronbach’s Alpha
NC1	Nation, consumers, and civilization* *		0.382	0.795**	
			1	*	
NC2				0.932**	
			0.666	*	
NC9				0.752**	
		0.773	0.441	*	
NC9		1		0.987**	0.795
			0.64	*	
MG1	Method and Guidance			0.822**	
			0.411	*	
MG2		0.865		0.796**	
		4	0.8841	*	0.932
MG9				0.931**	
			0.666	*	
AP1	Authority and Procedure			0.594**	
			0.404	*	
AP2				0.927**	
			0.854	*	
AP9				0.879**	
			0.743	*	
AP9				0.795**	
			0.8849	*	
SI1	Supervision Involvement			0.723**	
			0.362	*	
SI2		0.697		0.697**	
		2	0.521	*	0.769
SI9				0.976**	
			0.7693	*	
SI9				0.923**	
			0.574	*	
MD1	Modernization and Decision Making			0.843**	
			0.442	*	
MD2				0.976**	
		0.694	0.8754	*	0.943
MD9		6		0.765**	
			0.4932	*	
MD9				0.743**	
			0.759	*	

Note: Source: Field Survey.

The comparative fit index (CFI) assesses how well the research model matches the null model, implying no interactions between model components. As indicated in Table 7, the CFI value for all constructs is more than 0.89, suggesting that the measurement model is well-fit. The CFI number indicates that the model is well-fitting. The Composite Reliability method examines the internal consistency invariance of each construct using an observable variable and a concealed component. Exploratory Factor Analysis (EFA) Explanatory Factor Analysis (EFA) is a multivariate mathematical technique that has fundamentally created and proven hypotheses and measures. The EFA is a multivariate statistical approach that aims to categorize the fewest number of latent variables possible while still economically recounting the co-variation of computed parameters [46].

### 4.4 KMO and Bartlett’s test

Table 8 shows the data validity and dependability; the analysis KMO and Bartlett Test is provided below. The KMO is 0.897 > 0.5, and the BTS is 0.00, 0.001, indicating that data reliability and validity are unaffected.

**Table 8:** The results of KMO and Bartlett’s test.

Kaiser-Meyer-Olkin Measure of Sampling Adequacy.	0.897
Approx. Chi-Square	7326.451
Bartlett’s Test of Sphericity Df	525
Sig.	0

Source: Field Survey

### 4.5 Variables and component analysis

The total variance explained is shown in the table below, with six variables or components retrieved from SPSS. The total percentage is 84.2%, implying that the six variables account for 67% of the variation. [47] described the six components defined 75.5% of the overall variance. However, our analysis revealed 84.6% variance, indicating that the conflict is sufficient for our research. The six variables have to modify the item plot variables’ variance-covariance matrix to their respective causes. Table 9 shows the six latent variables were created using commonalities across the 0.5 scales to fit the variance-covariance matrix of the indicator variables.

**Table 9:** Results of component and variables analysis.

Component	Initial Eigenvalues			Extraction Sums of Squared Loadings			Rotation Sums of Squared Loadings		
		% of	Cumulative		% of	Cumulative		% of	Cumulative
	Total	Variance	%	Total	Variance	%	Total	Variance	%
First	11.537	42.467	42.467	11.537	42.467	42.467	3.768	13.457	13.457
Second	4.190	11.276	48.716	4.190	11.276	48.716	4.321	14.009	27.197
Third	4.322	9.453	63.332	4.322	9.453	63.332	4.156	13.221	2.492
Forth	2.492	8.212	67.366	2.492	8.212	67.366	3.264	12.876	51.064
Fifth	1.283	4.583	71.342	1.283	4.583	71.342	4.297	12.345	63.119
Sixth	1.246	4.223	84.225	1.246	4.223	84.225	3.067	10.978	84.225

Source: field survey

In the table above, the total variable’s extraction value is more significant than 0.5, suggesting that the whole variable meets the extractive collective’s requirement. Table 10 shows all indicators’ results with a loading factor greater than 0.5, and all of the hands were valid for calculating these latent variables.

**Table 10:** Initial and extraction result.

Indicator	Initial	Extraction	Indicator	Initial	Extraction
NC1	1	0.823	AP1	1	0.734
NC2	1	0.854	AP2	1	0.729
NC3	1	0.798	AP3	1	0.832
NC4	1	0.823	AP4	1	0.632
NC5	1	0.926	AP5	1	0.732
MG1	1	0.932	MD1	1	0.743
MG2	1	0.893	MD2	1	0.795
MG3	1	0.821	MD3	1	0.784
MG4	1	0.843	MD4	1	0.763

Source: field survey

### 4.6 Confirmatory Factor Analysis (CFA)

LISREL 8.80 was also used as part of confirmatory factor analysis. The CFA assessed the calculation model, which means that connections between manifest and latent variables are defined, and all latent variables are allowed. The component loads of the manifest variables about their latent variables are shown in the table below. In CFA, many statistical tests are required to assess how well the model fits the data. We have ensured that the model and the data don’t always match perfectly; implying that the model is "suitable" or even explaining a significant portion of the covariance and "excellent model fit" indicates the model implementation and results are presented in Table 11.

**Table 11:** Positive features assessment.

Fit Indicates	Model values	Acceptable values	Judgment of model fit
CMINDF	4.234	< 4.90	Excellent
RMR	0.063	< 0.07	Excellent
GFI	0.937	> 0.89	Agreed
CFI	0.793	> 0.75	Agreed
TLI	0.765	< 0.83	Agreed
IF	0.907	> 0.87	Agreed
RMSEA	0.08	≤ 0.08	Agreed

Source: field survey

Several fair indicator values, such as CMINDF, RMR, GFI, CFI, IFI, TLI, and RMSEA, are compared with the findings of the variables in the confirmatory study. The table shows that latent variables, i.e., GFI, CFI, TLI, and IFI, have acceptable values, and CMINDF and RMR meet the fit requirements. The selected model is ideal for the research. When is a confirmatory factor review performed? GFI = 0.923, CFI = 0.951, TLI = 0.936, IFI = 0.952, PCFI = 0.724, PGFI = 0.615, RMSEA = 0.064, and AIC = 141.163) are the consistency adaption values in the Measurement Model (MM), reliability, and validity.

#### 4.6.1 Analysis of mediation of SOBEL test

When we choose Table 12 as the target variable, the relationship becomes more solid. We find no link between administrative experience and success. Exporters make more excellent progress, but only in a small fraction of the actions.

**Table 12:** Result of indirect affect and SOBEL-test examining the mediating relationship.

			Mediating Effect Test		Test Statistics	P-value
			*b*	*S*_*b*_		
**NC**	**A**	0.798	0.324	0.085	1.679	0.00***
** **	Sa	0.083				
**SL**	**A**	0.876	0.632	0.643	0.083	6.214
** **	Sa	0.087				
**MI**	**A**	0.765	0.865	0.092	8.954	0.00***
	Sa	0.054				

The SOBEL-test, a method of determining the mediation impact among measuring variables, is used in our research to determine whether or not a mediation connection exists between the dependent and independent variables. In other words, the inclusion of a mediating variable in a SOBEL test assures that its influence is relevant when the dependent and independent variables are compared. Because the p-values are less than 0.01 (i.e., 0.00***), the result suggests that there is a partial mediation link between Technology modernization choice (TDC), Management Involvement (MI), and Independent factors.

### 4.6.2 Hypothesis test result

In any event, the societal cost of creative transformation is high. For example, changes in the labor market due to new skill requirements in the economy can result in skills gaps and skills oldness. The resulting in unemployment and enraptured labour markets; new items, like the arrangement of administrations can affect daily utilization propensities and decisions are shown in Table 13.

**Table 13:** Hypothesis testing results.

Hypothesis	Structural Path	P-value	Significance	Test result
1st	MDM→NCC	0	Significant	Rejected
2nd	MDM→SAL	0	Significant	Rejected
3rd	MDM→GOR	0	Significant	Rejected
4th	GI→TDA,PCC	0	Significant	Rejected
5th	GI→TDA,SAL	0	Significant	Rejected
6th	GI→TDA,GOR	0	Significant	Rejected

In the above table, all the P-values are less than 0.05, i.e., 0.00, which means there is a significant relationship between dependent and independent variables. This result rejects the entire initial hypothesis.

A new type of business can affect unemployment and enraptured labour markets; when the financial framework begins interplay of variation to innovations that end in all sections of society, the social cost of the underlying shock will smooth out.

## 5. Conclusions and recommendations

China’s economy has progressed from high-speed growth to high-quality development, and an innovation-driven economy has emerged as the cornerstone to the country’s current stability. The study focuses on talent agglomeration in China’s manufacturing industries and innovative industry 4.0. It established three dimensions of evaluation indicators to evaluate the incentive factors of talent agglomeration and industry 4.0 in manufacturing industries by using data of 2017 to 2019. For the data analysis structured equation modeling test and mediating variable in a SOBEL test are applied. The results show that Industry 4.0 is the primary practical corridor to official and familiar in sequence substitute policy and collaboration for talent agglomeration on research projects. The results confirmed that the hypotheses associated between dependent and independent variables have acceptable values which fit criteria and results will assist managers and policymakers in spotting talent agglomeration activities implemented to increase manufacturing businesses’ readiness to reap the most benefits from Industry 4.0 adoption. According to the findings, Industry 4.0 has a significant positive impact on the talent agglomeration of managers, executives, high-tech officers a significant spatial correlation between innovation talent agglomeration and Industry 4.0. The results of SOBEL test, structured equation modeling, analytic hierarchy process revealed that industry 4.0 has a clear influence on the latent agglomeration.

### 5.1 Suggestions for future research

China’s industries are still falling behind in terms of technology adoption. Concerned parties should create a distinct framework for industrial technology adoption, making it easier for businesses to employ technology. Even though this research covers many issues, certain areas still need to be explored further to future research in other industrial sectors, the manufacturing industry.

## Supporting information

S1 File Unveiling Industry 4.0’s appeal: Exploring talent agglomeration with survey questionnaires. (DOCX)

S1 Appendix (DOCX)
