# Supplementary material for: Inducement factor of talent agglomeration in the manufacturing industrial sector: A survey on the readiness of Industry 4.0 adoption
Source: PLoS One. 2023 Oct 5;18(10):e0263783. doi: 10.1371/journal.pone.0263783 (PMC10553246; doi:10.1371/journal.pone.0263783)
Supplement: S1 File — (DOCX) [file pone.0263783.s001.docx]

**A Survey Questionnaire**

- * = This field is required
- 0 = very low
- 5 = very high

**Name ***

**Surname ***

**Email ***

**Company Name ***

**Industry ***

**Number of employees ***

**General Questions**

**Why you would like to test the digital maturity of your organisation? ***

**Have you heard of the term Industrie 4.0?**

**Are you looking for solution partners to help with your Digital Transformation? ***

**Strategy & Leadership**

**The organisation has a digital vision to transform because of the new market needs ***

| 1 | 2 | 3 | 4 | 5 |
| --- | --- | --- | --- | --- |

**Leadership has made an effort to translate the digital vision down to all levels of the organisation ***

| 1 | 2 | 3 | 4 | 5 |
| --- | --- | --- | --- | --- |

**There is a team dedicated to the organisation's digital transformation and change ***

| 1 | 2 | 3 | 4 | 5 |
| --- | --- | --- | --- | --- |

**There is a business area prioritised for digital investments ***

| 1 | 2 | 3 | 4 | 5 |
| --- | --- | --- | --- | --- |

**There is a separate budget allocated for adopting digital technologies ***

| 1 | 2 | 3 | 4 | 5 |
| --- | --- | --- | --- | --- |

**Customer Experience**

**The organisation understands how customer demands are changing in the market ***

| 1 | 2 | 3 | 4 | 5 |
| --- | --- | --- | --- | --- |

**The organisation experiments with multiple digital channels to engage the customers ***

| 1 | 2 | 3 | 4 | 5 |
| --- | --- | --- | --- | --- |

**Digital technology is used to stay in touch with the customers and to solve their challenges ***

| 1 | 2 | 3 | 4 | 5 |
| --- | --- | --- | --- | --- |

**Data inputs from customer usage are used continuously for improving solutions and services ***

| 1 | 2 | 3 | 4 | 5 |
| --- | --- | --- | --- | --- |

**The organisation is able to offer customised solutions to capture higher share of the market segment ***

| 1 | 2 | 3 | 4 | 5 |
| --- | --- | --- | --- | --- |

**Operations**

**All production equipment is connected and real time data is available for decision making ***

| 1 | 2 | 3 | 4 | 5 |
| --- | --- | --- | --- | --- |

**There is one integrated platform which provides complete visibility and can be accessed by multiple users ***

| 1 | 2 | 3 | 4 | 5 |
| --- | --- | --- | --- | --- |

**It is possible to access all production information remotely ***

| 1 | 2 | 3 | 4 | 5 |
| --- | --- | --- | --- | --- |

**KPIs are well defined across functions and get updated automatically ***

| 1 | 2 | 3 | 4 | 5 |
| --- | --- | --- | --- | --- |

**Departments are able to collaborate easily through digital channels ***

| 1 | 2 | 3 | 4 | 5 |
| --- | --- | --- | --- | --- |

**Products & Innovations**

**The organisation is able to innovate rapidly as per the changing market requirements ***

| 1 | 2 | 3 | 4 | 5 |
| --- | --- | --- | --- | --- |

**The organisation leverages on digital technology for new product innovations (3D Printing, Virtualization) ***

| 1 | 2 | 3 | 4 | 5 |
| --- | --- | --- | --- | --- |

**New service models, enabled by digital technology, have been introduced ***

| 1 | 2 | 3 | 4 | 5 |
| --- | --- | --- | --- | --- |

**It is possible to analyse product usage information based on real time data streaming ***

| 1 | 2 | 3 | 4 | 5 |
| --- | --- | --- | --- | --- |

**Governance and risk strategy is in plan for Connected Products environment ***

| 1 | 2 | 3 | 4 | 5 |
| --- | --- | --- | --- | --- |

**People**

**A team of digital experts has been deployed to drive digital adoption across the organisation ***

| 1 | 2 | 3 | 4 | 5 |
| --- | --- | --- | --- | --- |

**Employees are able to leverage on digital tools for collaboration and remote connectivity ***

| 1 | 2 | 3 | 4 | 5 |
| --- | --- | --- | --- | --- |

**Ideas of digital transformation by employees are encouraged ***

| 1 | 2 | 3 | 4 | 5 |
| --- | --- | --- | --- | --- |

**Digital tools are used for knowledge management and skill enhancement ***

| 1 | 2 | 3 | 4 | 5 |
| --- | --- | --- | --- | --- |

**What worries you about Industrie 4.0 technologies? Is there anything else you would like to let us know?**

| 1 | 2 | 3 | 4 | 5 |
| --- | --- | --- | --- | --- |
