# Supplementary material for: Inducement factor of talent agglomeration in the manufacturing industrial sector: A survey on the readiness of Industry 4.0 adoption
Source: PLoS One. 2023 Oct 5;18(10):e0263783. doi: 10.1371/journal.pone.0263783 (PMC10553246; doi:10.1371/journal.pone.0263783)
Supplement: S1 Appendix — (DOCX) [file pone.0263783.s002.docx]

## Appendixes

**Abbreviations of the important terms**

CPS = Cyber-physical system

IOT = Internet of Things

IIOT = Industrial Internet of Things

CCAI = Cognitive Computing and Artificial Intelligence

EFA = Exploratory factor analysis

CFA = Confirmatory factor analysis

PA = Path Analysis

MA = Mediation Analysis

SEM = Structured Equation Modeling

AHP = Analytic Hierarchy Process

| **Table.14.** The Measurement Items of Constructs | | | |
| --- | --- | --- | --- |
| **Construct** | **Items** | **Observed Variables** | **Descriptions** |
| Peoples, Customers, and Culture | *NC1* | Use of client data | The company uses customers' input to create intelligent goods. |
|  | *NC2* | computerization of transactions | For buyers/customers, the organization is digitizing sales/services. |
|  | *NC3* | Costumer’s Digital media competence | Customers have a good understanding of digital media. |
|  | *NC4* | Value of ICT in company |  |
| Strategy and Leadership | *MG1* | Implementation I40 roadmap | In the workplace, organizations place a great value on information and communication technology (ICT). |
|  | *MG2* | Available resources for realization | The organization uses a route map. |
|  | *MG3* | Adaption of business models |  |
| Governance and Operations | *AP1* | Decentralization of processes | Enterprise-wide planning of Industry 4.0 initiatives |
|  |  |  | The organization has the resources necessary to implement Industry 4.0. |
|  | *AP3* | Interdepartmental collaboration |  |
|  | *AP4* | Labor regulations for I40 | Organizations are adapting their business models to accommodate industry 4.0 activities. |
| Government Intervention | *SI1* | financial support | For the adoption of new industrial technologies, organizational activities are dispersed. |
|  | *SI2* | Encouragement | Organizations use modeling and simulation. |
|  | *SI3* | Pieces of training and workshops | to use management or technical decision-making |
|  | *SI4* | Environmental regulation |  |
| Technology Innovation Decision Making | *MD1* | Top-level supportiveness | To assist Industry 4.0, the organization has an interdisciplinary nature. |
|  | *MD2* | Interest of managers | Labor rules are in place at the organization. |
|  | *MD3* | R&D activities |  |
|  | *MD4* | Organization culture | for the installation of I40 |
